# Sex-specific increase in susceptibility to metabolic syndrome in adult offspring after prenatal ethanol exposure with post-weaning high-fat diet

**DOI:** 10.1038/srep17679

**Published:** 2015-12-03

**Authors:** Zheng He, Jing Li, Hanwen Luo, Li Zhang, Lu Ma, Liaobin Chen, Hui Wang

**Affiliations:** 1 Department of Pharmacology, Basic Medical School of Wuhan University, Wuhan 430071, China; 2 Department of Orthopedic Surgery, Zhongnan Hospital of Wuhan University, Wuhan 430071, China; 3 Hubei Provincial Key Laboratory of Developmentally Originated Disease, Wuhan 430071, China; 4 Department of Epidemiology & Health Statistics, Public Health School of Wuhan University, Wuhan 430071, China

## Abstract

Prenatal ethanol exposure (PEE) is an established risk factor for intrauterine growth retardation. The present study was designed to determine whether PEE can increase the susceptibility of high-fat diet (HFD)-induced metabolic syndrome (MS) in adult offspring in a sex-specific manner, based on a generalized linear model analysis. Pregnant Wistar rats were administered ethanol (4 g/kg.d) from gestational day 11 until term delivery. All offspring were fed either a normal diet or a HFD after weaning and were sacrificed at postnatal week 20, and blood samples were collected. Results showed that PEE reduced serum adrenocorticotropic hormone (ACTH) and corticosterone levels but enhanced serum glucose, insulin, insulin resistant index (IRI), triglyceride and total cholesterol (TC) concentrations. Moreover, the analysis showed interactions among PEE, HFD and sex. In the PEE offspring, HFD aggravated the decrease in ACTH and corticosterone levels and further increased serum glucose, insulin, triglyceride and TC levels. The changes of serum ACTH, glucose and IRI levels in the female HFD rats were greater than those in the male HFD rats. Our findings suggest that PEE enhances the susceptibility to MS induced by HFD in a sex-specific manner, which might be primarily associated with the neuroendocrine metabolic programming by PEE.

Metabolic syndrome (MS), referring to a clustering of metabolic risk factors including central obesity, glucose intolerance, dyslipidemia and hypertension, is becoming one of the main threats to human health worldwide^1^. The prevalence of MS is increasing annually^2^. In United States, the morbidity rates associated with MS are 9.2% in adolescents^3^ and 22.9% in adults^4^. Intrauterine growth retardation (IUGR), defined as a birth weight below the 10th customized centile for gestational age, is characterized primarily by the poor growth potential of a fetus *in utero*^5^. In an epidemiological survey, the prevalence of overweight and obesity was high in adult women who were *in utero* during the Dutch or Chinese famine^6^, which also led to a higher risk of MS^7^. Epidemiological investigations^8^,^9^ and animal studies^10^,^11^ found that fetuses born with IUGR had a higher risk of developing MS and cardiovascular disease in adulthood, and that the incidence of MS in adults who previously experienced IUGR was 5.75-fold higher than that in healthy adults^9^. However, the underlying mechanisms are still unclear. To date, the most acceptable theory is the ‘thrifty phenotype hypothesis’^12^, which proposes that exposure of a fetus to an adverse intrauterine environment increases the sensitivity of the peripheral tissues to metabolic hormones (e.g., glucocorticoid), the increased sensitivity improves the fetal survival rate. The offspring will exhibit catch-up growth and have a higher risk of developing MS in adulthood as the nutritional pattern improves after birth^13^.

Prenatal ethanol exposure (PEE) is an established risk factor for several adverse birth outcomes, including multiple birth defects, mental retardation and IUGR, which are collectively known as fetal alcohol effects^14^,^15^. Many studies have shown that PEE induces IUGR^16^,^17^, which may be associated with PEE-impaired placentation^16^. Such IUGR offspring may present catch-up growth and develop dyslipidemia and hyperglycemia, showing increased susceptibility to MS in adulthood^13^,^18^. High-fat diet (HFD) is one of the main environmental factors accounting for the incidence of MS^19^. Epidemiological survey found ethanol exposure during pregnancy increases obesity rate and fat accumulation after birth^20^. Meanwhile, many animal studies have also found the ethanol-induced fat infiltration widely exists in pancreas, liver and subcutaneous fat^21^,^22^,^23^. Our previous studies confirmed that PEE increases the susceptibility to non-alcoholic fatty liver disease induced by a HFD in rat offspring^23^, which may be associated with fetal over-exposure to maternal glucocorticoid, resulting in both functional inhibition of the hypothalamic–pituitary–adrenal (HPA) axis^24^ and insulin-like growth factor 1-associated glucose and lipid metabolic alteration in peripheral tissues^23^. Furthermore, because these changes persist after birth, they manifest as HPA axis dysfunction and glucocorticoid-dependent blood glucose and lipid metabolic alterations in adult rats^25^.

Study showed that there were sex-specific changes in the key factors regulating glucose and lipid metabolism, such as insulin and adiponectin signaling pathways, which indicates different effects of the HFD on male and female rats^26^. Overall, PEE, HFD and sex are likely to increase the risk of MS development in IUGR offspring, however, the interactions among these three factors are unclear. In the present study, a rat IUGR model was established by PEE as previously described^23^, and a post-weaning HFD was used to induce MS in the offspring. Firstly, we observed alterations in HPA axis activity and glucose and lipid metabolism, including changes in blood adrenocorticotropic hormone (ACTH), corticosterone, glucose and lipid levels. Then, the interactions among PEE, HFD and sex were studied based on a generalized linear model. This study aimed to explore the risk factors for adult MS characterized by intrauterine origin and the links among them, which will be beneficial for elucidating the underlying mechanisms responsible for the susceptibility of IUGR offspring to adult MS and associated diseases. This study may also deepen our understanding of sex-specific differences in MS susceptibility of PEE rat offspring fed a HFD.

## Results

### Comparison between groups —Least significant difference (LSD) *post hoc* test Alterations in HPA axis activities

**Figure 1:**
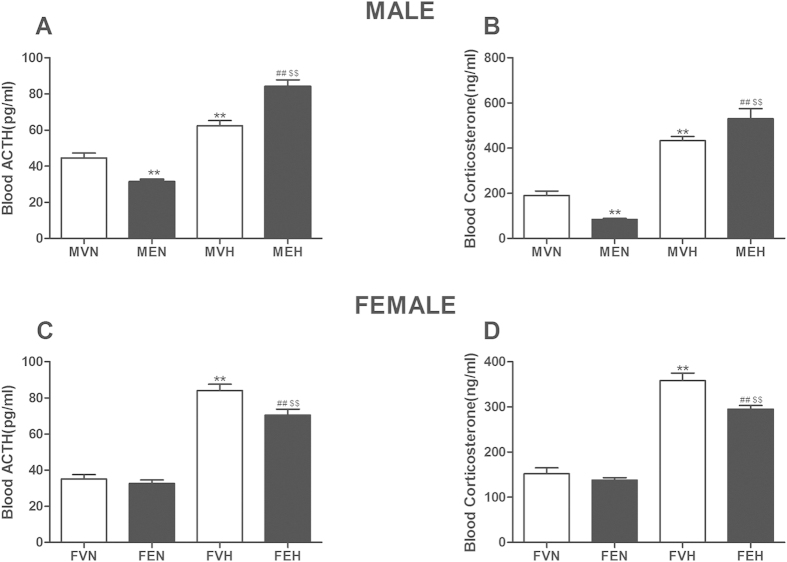
Effects of prenatal ethanol (4 g/kg.d) exposure on serum adrenocorticotrophic hormone (ACTH) and corticosterone concentrations in the rat offspring. (**A**) Male ACTH concentration; (**B**) Male corticosterone concentration; (**C**) Female ACTH concentration; (**D**) Female corticosterone concentration. Mean ± S.E.M., n = 8. ^**^P < 0.01 MVN *vs*. MEN and MVH *vs* MEH or ^**^P < 0.01 FVN *vs*. FEN and FVH *vs* FEH; ^##^*P* < 0.01 MVN *vs*. MVH and MEN *vs* MEH or ^##^*P* < 0.01 FVN *vs*. FVH and FEN *vs* FEH. MVN: male offspring by prenatal vehicle exposure with normal diet; MEN: male offspring by prenatal ethanol exposure with normal diet; MVH: male offspring by prenatal vehicle exposure with high-fat diet (HFD); MEH: male offspring by prenatal ethanol exposure with HFD; FVN: female offspring by prenatal vehicle exposure with normal diet; FEN: female offspring by prenatal ethanol exposure with normal diet; FVH: female offspring by prenatal vehicle exposure with HFD; FEH: female offspring by prenatal ethanol exposure with HFD.

For the male offspring (as shown in Fig. 1A,B), the serum ACTH and corticosterone concentrations were lower in the male offspring by prenatal ethanol exposure with normal diet (MEN) group (*P* < 0.01) than in the male offspring by prenatal vehicle exposure with normal diet (MVN) group, and both concentrations were higher in the male offspring by prenatal ethanol exposure with HFD (MEH) group than in the male offspring by prenatal vehicle exposure with HFD (MVH) group (*P* < 0.01). In the MVH group, the serum ACTH and corticosterone levels were higher than in the MVN group *(P* < 0.01), and both parameters were higher in the MEH group than in the MVH group (*P* < 0.01).

For the female offspring (as shown in Fig. 1C,D), there were no significant differences between the female offspring by prenatal vehicle exposure with normal diet (FVN) and female offspring by prenatal ethanol exposure with normal diet (FEN) groups in terms of the serum ACTH and corticosterone levels, and the serum ACTH and corticosterone levels were lower in the female offspring by prenatal ethanol exposure with HFD (FEH) group than in the female offspring by prenatal vehicle exposure with HFD (FVH) group (*P* < 0.01). Meanwhile, the serum ACTH and corticosterone levels were higher in the FVH group than in the FVN group (*P* < 0.01), and both parameters were higher in the FEH group than in the FEN group (*P* < 0.01).

### Alterations in serum glucose and insulin levels

**Figure 2:**
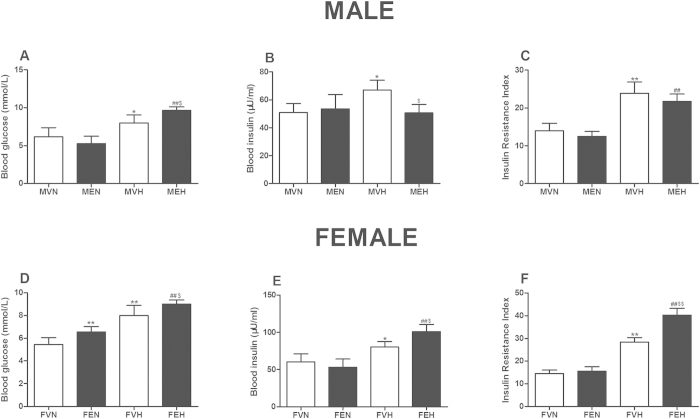
Effects of prenatal ethanol (4 g/kg.d) exposure on serum glucose, serum insulin concentrations and insulin resistance index (IRI) in the rat offspring. (**A**) Male glucose concentration; (**B**) Male insulin concentration; (**C**) Male IRI; (**D**) Female glucose concentration; E: Female insulin concentration; F: Female IRI. Mean ± S.E.M., n = 8. ^*^P < 0.05, ^**^P < 0.01 MVN *vs*. MEN and MVH *vs* MEH or ^*^P < 0.05, ^**^P < 0.01 FVN *vs*. FEN and FVH *vs* FEH; ^#^*P* < 0.05, ^##^*P* < 0.01 MVN *vs*. MVH and MEN *vs* MEH or ^#^*P* < 0.05, ^##^*P* < 0.01 FVN *vs*. FVH and FEN *vs* FEH. MVN: male offspring by prenatal vehicle exposure with normal diet; MEN: male offspring by prenatal ethanol exposure with normal diet; MVH: male offspring by prenatal vehicle exposure with high-fat diet (HFD); MEH: male offspring by prenatal ethanol exposure with HFD; FVN: female offspring by prenatal vehicle exposure with normal diet; FEN: female offspring by prenatal ethanol exposure with normal diet; FVH: female offspring by prenatal vehicle exposure with HFD; FEH: female offspring by prenatal ethanol exposure with HFD.

For the male offspring (as shown in Fig. 2A/B/C), there were no differences between the MVN and MEN groups in terms of the serum glucose and insulin levels or the insulin resistance index (IRI). However, the serum glucose concentration in the MEH group was markedly higher than that in the MVH group, and the serum insulin concentration in the MEH group was markedly lower than that in the MVH group (*P* < 0.05). Meanwhile, the serum glucose level, serum insulin concentration and IRI were higher in the MVH group than in the MVN group (*P* < 0.05, *P* < 0.01), and the serum glucose and IRI levels were higher in the MEH group than in the MEN group (*P* < 0.01).

For the female offspring (as shown in Fig. 2D/E/F), the serum glucose level in the FEN group was higher than that in the FVN group (*P* < 0.01), and the serum glucose level, insulin level and IRI were higher in the FEH group than in the FVH group (*P* < 0.05, *P* < 0.01). Meanwhile, the serum glucose level, insulin level and IRI in the FVH group were higher than those in the FVN group (*P* < 0.05, *P* < 0.01), and the serum glucose level, insulin level and IRI in the FEH group were higher than those in the FEN group (*P* < 0.01).

### Alterations in serum triglyceride and TC levels

**Figure 3:**
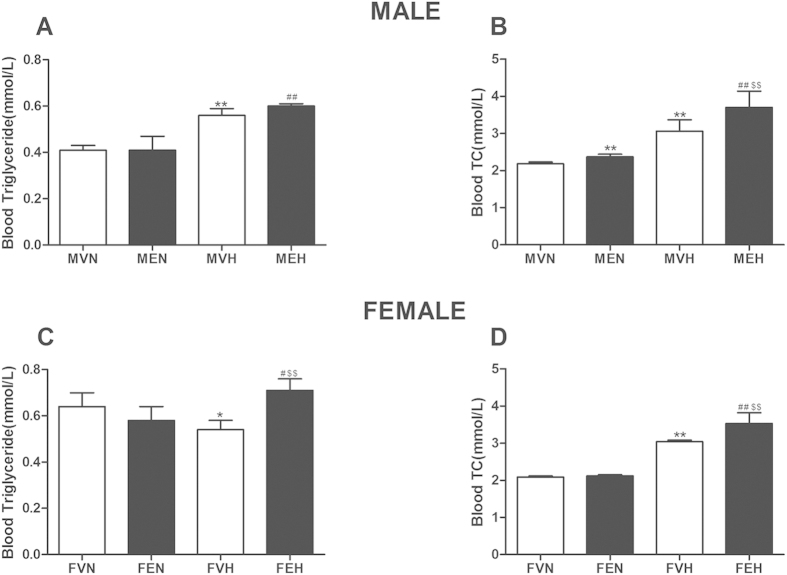
Effects of prenatal ethanol (4 g/kg.d) exposure on serum triglyceride and cholesterol (TC) concentrations in the rat offspring. (**A**) Male triglyceride concentration; (**B**) Male TC concentration; (**C**) Female triglyceride concentration; (**D**) Female TC concentration. Mean ± S.E.M., n = 8. ^**^P < 0.01 MVN *vs*. MEN and MVH *vs* MEH or ^**^P < 0.01 FVN *vs*. FEN and FVH *vs* FEH; ^#^*P* < 0.05, ^##^*P* < 0.01 MVN *vs*. MVH and MEN *vs* MEH or ^#^*P* < 0.05, ^##^*P* < 0.01 FVN *vs*. FVH and FEN *vs* FEH. MVN: male offspring by prenatal vehicle exposure with normal diet; MEN: male offspring by prenatal ethanol exposure with normal diet; MVH: male offspring by prenatal vehicle exposure with high-fat diet (HFD); MEH: male offspring by prenatal ethanol exposure with HFD, FVN: female offspring by prenatal vehicle exposure with normal diet; FEN: female offspring by prenatal ethanol exposure with normal diet; FVH: female offspring by prenatal vehicle exposure with HFD; FEH: female offspring by prenatal ethanol exposure with HFD.

For the male offspring (as shown in Fig. 3A,B), the serum TC concentration was higher in the MEN and MEH groups than in the MVN and MVH groups, respectively (*P* < 0.01). Meanwhile, the serum triglyceride and TC concentrations were significantly higher in the MVH and MEH groups than in the MVN and MEN groups, respectively (*P* < 0.01).

For the female offspring (as shown in Fig. 3C,D), between the FVN and FEN groups, there were no significant differences in the serum triglyceride and TC levels, and the serum triglyceride and TC concentrations were higher in the FEH group than in the FVH group (*P* < 0.01). Meanwhile, the serum TC level was higher in the FVH group than in the FVN group (*P* < 0.01), and the serum triglyceride and TC concentrations were higher in the FEH group than in the FEN group (*P* < 0.05, *P* < 0.01).

### Interactions among PEE, HFD and sex: — generalized linear model analysis Interaction in serum ACTH and corticosterone concentrations

With respect to serum ACTH level (Table 1), PEE groups were lower than in the vehicle groups (test substances *P* < 0.05), and HFD groups were higher than normal diet groups (Diet *P* < 0.01). There were effects in the PEE*HFD, HFD*sex and PEE*sex (*P* < 0.01), which manifested as PEE decreased serum ACTH level while HFD has the opposite effect; and serum ACTH level of female offspring was increased more obviously than that of males in the HFD groups; while in the PEE groups, the female serum ACTH level was decreased more significantly than the male. Moreover, there was an interaction in PEE*HFD*sex (*P* < 0.01).

With respect to serum corticosterone levels (Table 1), when compared to normal diet, it was increased in HFD groups (Diet *P* < 0.01), and the female groups were lower than male groups (Sex *P* < 0.01). There were interactions in PEE*HFD and HFD*sex (*P* < 0.01), which indicated that PEE*HFD and HFD*sex decreased serum corticosterone level. Moreover, there was an interaction in PEE*HFD*sex (*P* < 0.01).

### Interaction in serum glucose and insulin concentrations

The results of glucose levels (Table 1) showed that, PEE groups were higher than vehicle groups (*P* < 0.01), and HFD groups were higher than normal diet ones (*P* < 0.01). There were interactions in PEE*HFD and PEE*sex (*P* < 0.05, *P* < 0.01), where PEE*HFD decreased serum glucose level; and this decrease was more obvious in female offspring than in the males. Moreover, there was an interaction in PEE*HFD*sex (*P* < 0.01).

With respect to serum insulin level (Table 1), PEE groups were lower than vehicle groups (Test substances *P* < 0.05), and HFD groups were higher than normal diet groups (Diet *P* < 0.01). There were interactions in PEE*HFD (*P* < 0.01), where PEE*HFD increased serum insulin level. Moreover, there was an interaction in PEE*HFD*sex (*P* < 0.05), the serum insulin level was increased more obviously in the female than that of the male in the PEE offspring with HFD.

### Interaction in serum triglyceride and TC concentration

For the serum triglyceride level (Table 1): PEE groups were significantly higher than vehicle groups (*P* < 0.05), HFD groups were also higher than normal diet groups (*P* < 0.01). Female groups were higher than male groups (*P* < 0.01). There were interactions in PEE*HFD and HFD*sex (*P* < 0.05, *P* < 0.01), where PEE*HFD and HFD*sex both aggravated the increase in serum triglyceride level. Moreover, there was an interaction in PEE*HFD*sex (*P* < 0.01).

For the serum TC level (Table 1): PEE groups were higher than vehicle groups (*P* < 0.01), while compared with normal diet, HFD groups were significantly lower (*P* < 0.01), and female groups had an decreasing trend than male groups (*P* = 0.06). There was an interaction in PEE*HFD (*P* < 0.01), reflecting by PEE*HFD aggravated the increase of serum TC level.

## Discussion

It is known that PEE, HFD and sex play important roles in the fetal origin of MS^23^,^27^,^28^. PEE can enhance the susceptibility to MS in female adult offspring^23^, and a HFD can lead to obesity, insulin resistance (IR) and other metabolic diseases^29^,^30^. Early metabolic programming can contribute to the variability observed in response to nutritional interventions and can further the development of metabolic diseases in later life, particularly in environments in which a HFD is prevalent^31^. In this study, we found that PEE increased the serum glucose and TC levels, and a HFD increased the levels of all blood phenotypes except for triglycerides. Sex-specific differences were observed in all blood phenotypes, with the exception of serum ACTH, insulin and glucose levels. Therefore, we speculated that PEE, HFD and sex are important factors in the developmental origins of fetal-originated MS.

In the present study, based on generalized linear model analysis, we observed the interaction of the combination of PEE and HFD-decreased serum ACTH, corticosterone and glucose levels, with enhanced serum insulin, triglyceride and TC levels. Additionally, compared with the MVH and FVH groups, the serum ACTH, corticosterone, glucose, triglycerides and TC levels were significantly increased in the MEH and FEH groups, respectively. These results suggest that combined with a HFD, the effects of PEE on serum ACTH and corticosterone may be related to the basal activity of the HPA axis, which may be increased by a HFD to promote the synthesis and secretion of hypothalamic corticotrophin-releasing hormone (CRH) in PEE offspring^32^. Based on the HPA axis-associated neuroendocrine metabolic programming mechanism through PEE^33^, we expected PEE to reduce serum triglyceride and TC levels with the increased activity of the HPA axis; however, the levels of serum triglyceride and TC were enhanced in the present study, probably due to the HFD *per se* (which contained 11.5% lard and 0.5% cholesterol)^34^. Studies have shown that a HFD increases serum insulin levels^35^, and PEE decreases these levels by damaging pancreatic β cells^36^. Here we found that PEE decreased serum insulin levels and a HFD increased these levels, which is consistent with the above-mentioned studies (Table 1), however, the interaction between PEE and HFD increased the blood insulin concentration, which suggested that the PEE offspring fed by HFD may develop insulin resistance.

Studies have identified sex disparities in the amygdalar neuronal plasticity of adult rats exposed to ethanol^37^. The interaction between sex and PEE was also investigated in the present study. We found that in the females, the serum glucose level was increased in the FEN group compared with the FVN group, whereas in the males, these levels were decreased in the MEN group compared with the MVN for the PEE offspring fed normal diets. Furthermore, there was an interaction between PEE and sex (not considering the HFD) based on the generalized linear model analysis, which primarily suggests that the levels of serum ACTH and glucose increased to a greater degree in the female PEE offspring. These results indicated that the combination of PEE and sex changed the HPA axis-associated neuroendocrine metabolic programming. The different sensitivities of the HPA axis in the female and male IUGR offspring rats may be involved in the observed sex differences^38^. The corticosterone feedback regulation (by binding to the glucocorticoid receptor) is an important mode of HPA axis regulation^39^, and there are sex differences in corticosterone actions^40^. Therefore, in terms of sex differences, corticosterone may play a role in the HPA axis. In addition, the corticosterone-related sex difference was probably caused by the regulation of sex hormones^41^ and the sex dependent expression of corticosterone metabolic enzymes^42^. Studies have shown that androgen and estrogen can affect glucose/lipid metabolism in peripheral organs (e.g., the liver) through differential regulation of the HPA axis^43^,^44^. Therefore, this sex disparity in metabolic programming by PEE was probably related to the effects of sex hormones on glucose and lipid metabolic functions.

According to other reports, sex disparities in emotional profiles and metabolic functionality have been found in the prenatal HFD-exposed mice offspring^45^, and a HFD can increase ovulatory dysfunction in postpubertal female rats, ultimately resulting in adverse metabolic and reproductive outcomes in female offspring^46^. In addition, HPA axis-associated neuroendocrine dysfunction induced by HFD is more severe in female rats when the glucocorticoid receptor expression levels are decreased in the hypothalamus, which suggests that the female is more sensitive to fat-induced nutritional imbalance^47^. In the present study, we found that, compared with the FVH group, serum triglyceride levels in the females demonstrated an increasing trend in the FVN group, whereas in the males, these levels were decreased in the MVN group comparing with the MVH group. Furthermore, there was an interaction between HFD and sex, which decreased the serum ACTH levels in the male offspring rats and increased the serum corticosterone and triglyceride levels in the female offspring rats. In the HFD groups, the decreased serum ACTH level and the increased serum corticosterone and triglyceride levels in the females were much more remarkable than those in the males. These results suggested that HFD-induced alteration of the HPA axis function was characterized by significant sex differences.

Few studies have explored the interactions between PEE, HFD and sex. In the present study, based on the generalized linear model analysis, the interaction between PEE and HFD increased the serum ACTH, corticosterone, insulin, and glucose levels in the female offspring and decreased the serum triglyceride levels in the male offspring, which suggests that PEE enhanced the susceptibility to MS in the adult female offspring fed a post-weaning HFD. In addition, we found that serum glucose levels increased but corticosterone levels decreased in the PEE female offspring fed a HFD. In terms of how reduced serum cortisol/ACTH levels increased the serum glucose levels in the female offspring in this study, we found that the serum corticosterone and glucose levels were higher in the HFD group than in the rats fed a normal diet, without sex differences observed, which is consistent with other reports^48^,^49^. Meanwhile, it has been shown that fetal pancreas development is damaged in the PEE offspring rats, which can lead to increased serum glucose levels at 6 months of age^50^. In the present study, we observed decreased serum corticosterone levels and increased glucose levels in the rats fed HFDs, primarily in the PEE female offspring compared with the prenatal vehicle exposure female offspring. Therefore, this result may be caused by the combined effect of PEE and a HFD.

## Conclusions

In this study, we demonstrated that PEE enhanced the susceptibility to MS induced by HFD in a sex-specific manner, which was probably associated with neuroendocrine metabolic programming by PEE. Furthermore, several significant interactions existed among PEE, HFD and sex, which presented as HFD aggravated PEE-associated neuroendocrine metabolic programming with greater changes observed in females. This study may provide an experimental basis for confirming the impact factors (such as PEE, HFD and sex) and their interactions in adult MS, while expanding our understanding of the negative effects of nutritional intervention on PEE offspring. Increased knowledge of these subjects may be useful for preventing and/or retarding the development of adult metabolic diseases.

## Materials and Methods

### Chemicals and reagents

Ethanol (analytical purity) was obtained from Zhen Xin Co., Ltd. (Shanghai, China). Isoflurane was purchased from Baxter Healthcare Co. (Deerfield, IL, USA). The rat ACTH and insulin radioimmunoassay kits were obtained from Beijing North Biotech Institute (Beijing, China). The ELISA kit for the determination of the serum corticosterone concentration was purchased from Assaypro (St Charles, USA). The glucose oxidase assay kit was provided by Shanghai Mind Bioengineering Co., Ltd. (Shanghai, China). The triglyceride and total cholesterol (TC) assay kits were obtained from Sangon Biotech Co., Ltd. (Shanghai, China). All other chemicals and reagents were of analytical grade.

### Animals and treatment

The animal care protocol was approved by the Committee on the Ethics of Animal Experiments of the Wuhan University School of Medicine (Permit Number: 14016). Specific pathogen free (SPF) Wistar rats (females weighing 200–240 g; males weighing 260–300 g) were obtained from the Experimental Center of Hubei Medical Scientific Academy (No. 2008–0005, Hubei, China). Animal experiments were performed at the Center for Animal Experiment of Wuhan University (Wuhan, China), which is accredited by the Association for Assessment and Accreditation of Laboratory Animal Care International (AAALAC International). All animal experimental procedures were performed in accordance with the Guidelines for the Care and Use of Laboratory Animals of the Chinese Animal Welfare Committee and the International Council on Research Animal Care.

Animals were kept under temperature-controlled conditions on a 12 hour light: dark cycle with *ad libitum* access to standard chow and tap water at all time. After one week of acclimation, two females were mated with one male for one night. Upon confirmation of mating based on the appearance of sperm in a vaginal smear, the day was designated GD 0. Pregnant females were then transferred to individual cages.

Pregnant rats were randomly divided into two groups: control and PEE groups, and 8–10 dams were in each group. Starting from GD11 until term delivery (GD21), the PEE group was administered with oral gavage at a dose of 4 g/kg.d (36%, 14 ml/kg) ethanol, as previously described^51^. The control group was given the same volume of distilled water. Feed intake and weight gain of PEE-treated group during pregnancy were similar to control.

At parturition, the dams and their offspring were fed *ad libitum*. On postnatal day 1 (PD 1), the numbers of pups in each litter were selected to 8 pups randomly, to ensure adequate and standardized nutrition until weaning^47^. At PW 4, 32 pups from 8 different mothers were selected randomly for each group (16 male and 16 female IUGR pups from the ethanol group, 16 male and 16 female normal pups from the control group), and all pups were fed either a normal diet or a HFD *ad libitum* before being sacrificed. The standard rodent chow purchased from the Experimental Centre of Hubei Medical Scientific Academy contained 21% kcal from protein, 68.5% kcal from carbohydrate and 10.5% kcal from fat. The HFD was previously reported by our laboratory^49^ and contained 88.0% corn flour, 11.5% lard, and 0.5% cholesterol, which provided 18.9% kcal from protein, 61.7% kcal from carbohydrate and 19.4% kcal from fat.

At PW 20, all the rat offspring were fasted overnight after 8 p.m., then anesthetized with isoflurane and decapitated the next morning between 8 a.m. and 10 a.m. in a room separate from the room in which the other animals were kept. Trunk blood was collected and serum was prepared by centrifugation at 17,205 × g for 15 min at 4 °C. Serum samples were stored at −80 °C until they were used for the measurement of ACTH, corticosterone, glucose, insulin, triglyceride and TC concentrations.

### Study Design

**Figure 4:**
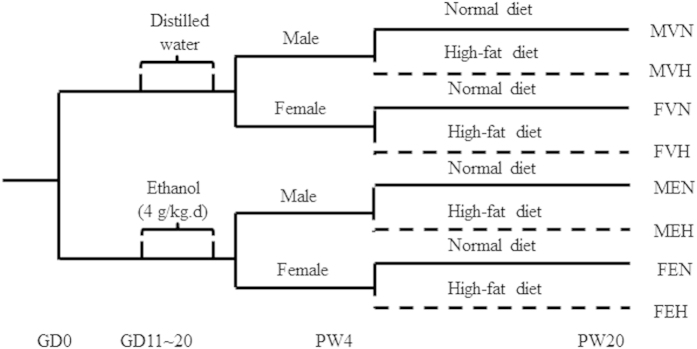
The schedule of animal treatment from gestation day 0 (GD 0) to postnatal week 20 (PW 20). MVN: male offspring by prenatal vehicle exposure with normal diet; MEN: male offspring by prenatal ethanol exposure with normal diet; MVH: male offspring by prenatal vehicle exposure with high-fat diet (HFD); MEH: male offspring by prenatal ethanol exposure with HFD; FVN: female offspring by prenatal vehicle exposure with normal diet; FEN: female offspring by prenatal ethanol exposure with normal diet; FVH: female offspring by prenatal vehicle exposure with HFD; FEH: female offspring by prenatal ethanol exposure with HFD.

A schematic representation of the procedure for maternal and offspring rat treatment was shown in Fig. 4. Naming principles are as follows: male offspring by prenatal vehicle exposure with normal diet (MVN), male offspring by prenatal ethanol exposure with normal diet (MEN), male offspring by prenatal vehicle exposure with HFD (MVH), male offspring by prenatal ethanol exposure with HFD (MEH), female offspring by prenatal vehicle exposure with normal diet (FVN), female offspring by prenatal ethanol exposure with normal diet (FEN), female offspring by prenatal vehicle exposure with HFD (FVH), female offspring by prenatal ethanol exposure with HFD (FEH).

### Analysis of blood samples

The concentrations of serum ACTH and corticosterone were measured via an isotope labeling kit and an ELISA kit, respectively, following the manufacturers’ protocols. The intra-assay coefficients of variation for ACTH and corticosterone determination were 10% and 15%, respectively, and the inter-assay coefficients of variation were 5.0% and 7.2%, respectively. The concentrations of serum glucose, triglyceride and TC were measured using the respective biochemical assay kits following the manufacturers’ protocols. Serum insulin was determined using a radioimmunoassay kit. The IRI were calculated as described below.


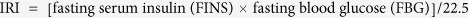


### Statistical analysis

SPSS 18 (SPSS Science Inc., Chicago, IL, USA) and Prism (GraphPad Software, La Jolla, CA, USA) were used for data analysis. All data are presented as the mean ± S.E.M. To ensure the results were described comprehensively, we used LSD *post hoc* test for statistical significance among multi-groups comparisons as others used^52^ to analyze the effect of ethanol exposure and diet in female and male rats respectively. For example, the effect of ethanol exposure was analyzed between MVN and MEN, and the effect of diet was analyzed between MVN and MVH.

Generalized linear model analysis was used to explore the interactions among PEE, diet and sex in the development of MS, including the main effects and interaction effects of two or three factors. Sex was coded as 1for female offspring and 0 for male offspring. The interaction means the combination effects of PEE and HFD, HFD and sex, PEE and sex, as well as PEE, HFD and sex, which were abbreviated as PEE*HFD, HFD*sex PEE*sex and PEE*HFD*sex, respectively. Meanwhile, we presented a table (Table 1) with the results of P values (representing statistical significance) and B values (i.e., the regression coefficient, which reflect the effects of interaction on the dependent variable) for the analyses. If the B value is positive, it suggests that the influence factors increase the change in the dependent variable; vice versa, the influence factors decrease the change of the dependent variable when the B value is negative.

## Additional Information

**How to cite this article**: He, Z. *et al*. Sex-specific increase in susceptibility to metabolic syndrome in adult offspring after prenatal ethanol exposure with post-weaning high-fat diet. *Sci. Rep*. **5**, 17679; doi: 10.1038/srep17679 (2015).

## Figures and Tables

**Table 1:** Interactional analysis with generalized linear model among ethanol, high-fat diet (HFD) and sex on neuroendocrine metabolism-related blood phenotypes in rat offspring.

Indexs	Factors							
	Sex		Ethanol	HFD	Ethanol *HFD	Ethanol *Sex	HFD* Sex	Ethanol *HFD*Sex
ACTH	B	9.49	−2.51	48.98	−11.12	−10.72	−31.25	46.13
	Sig	P > 0.05	P < 0.05	P < 0.01	P < 0.01	P < 0.01	P < 0.01	P < 0.01
Corticosterone	B	37.81	−14.75	205.4	−4.21	−90.90	37.70	189.21
	Sig	P < 0.05	P > 0.05	P < 0.01	P < 0.01	P > 0.05	P < 0.01	P < 0.05
Glucose	B	0.71	1.10	2.53	−0.09	−2.02	−0.65	2.33
	Sig	P > 0.05	P < 0.01	P < 0.01	P < 0.01	P < 0.05	P > 0.05	P < 0.01
Insulin	B	1.43	−3.10	42.23	10.05	−4.23	−0.65	12.50
	Sig	P > 0.05	P < 0.05	P < 0.01	P < 0.01	P > 0.05	P > 0.05	P < 0.05
Triglyceride	B	−0.23	−0.06	−0.07	0.20	0.05	0.21	−0.15
	Sig	P < 0.05	P < 0.05	P < 0.01	P < 0.05	P > 0.05	P < 0.01	P < 0.01
TC	B	0.09	0.02	0.95	0.47	0.17	0.08	0.03
	Sig	P < 0.1	P < 0.01	P < 0.01	P < 0.01	P > 0.05	P > 0.05	P > 0.05

ACTH: adrenocorticotropic hormone; B: regression coefficient. Sig: P value, TC: total cholesterol.
